# Swimming coaches’ professional development and training practices: an international survey

**DOI:** 10.3389/fspor.2023.1229066

**Published:** 2023-07-18

**Authors:** Athanasios A. Dalamitros, Alexandros Nikolopoulos, Katerina Varsamidou, Vassilios Gourgoulis, Aglaia Zafeiroudi, Andreas Loukovitis, Vicente Javier Clemente-Suárez, José Francisco Tornero-Aguilera, Cormac Powell

**Affiliations:** ^1^ Laboratory of Evaluation of Human Biological Performance, School of Physical Education & Sport Sciences, Aristotle University of Thessaloniki, Thessaloniki, Greece; ^2^ School of Physical Education & Sport Sciences, National and Kapodistrian University of Athens, Athens, Greece; ^3^ School of Physical Education & Sport Sciences, Democritus University of Thrace, Komotini, Greece; ^4^ Applied Leisure Sciences Laboratory, School of Physical Education & Sport Sciences, University of Thessaly, Trikala, Greece; ^5^ Laboratory of Human Studies and Sport Psychology, School of Physical Education & Sport Sciences, Aristotle University of Thessaloniki, Thessaloniki, Greece; ^6^ Faculty of Sports Sciences, Universidad Europea de Madrid, Madrid, Spain; ^7^ Grupo de Investigación en Cultura, Educación y Sociedad, Universidad de la Costa, Barranquilla, Colombia; ^8^ High-Performance Unit, Sport Ireland, Sport Ireland Campus, Dublin, Ireland; ^9^ Sport and Human Performance Research Centre, Health Research Institute, University of Limerick, Limerick, Ireland; ^10^ Physical Activity for Health Cluster, Health Research Institute, University of Limerick, Limerick, Ireland

**Keywords:** coaching, psycho-social issues, training approaches, professional background, competitive swimming

## Abstract

This study investigated swim coaches' perceptions of professional development issues and current training practices according to their coaching experience, education level, and gender. An online survey (January—April 2022) was completed by 123 swim coaches (96 male/27 female) of competitive swimmers based in 41 countries. The survey consisted of 36 questions divided into six sections: (1) background information, (2) developing swim coaching through learning, (3) self-evaluation, (4) interpersonal-intrapersonal interactions, (5) life skills, and (6) analyzing swimming performance. Pearson chi-square assessed the relationship between the frequency of responses and professional background and gender. The survey results indicated that swim coaches' educational level is potentially one of the most influential parameters affecting the coaches' perceptions about their own professional development. The data presented may be used for the future design of coach education programmes as they advance current knowledge on understanding psycho-social issues related to professional development and training perceptions involved in the competitive swimming environment.

## Introduction

Sports coaching is a complex and dynamic process that requires continuous professional development, considering the multidimensional and central role of the coach to facilitate training practice (1). In the competitive sporting environment, coaching is a demanding task involving the complex interaction between coaches' performance and athletes' short- and long-term support to reach their full athletic potential (2). In an attempt to support sports coaches' performance, formal (e.g., programs by national governing bodies), informal (e.g., experiential learning), and non-formal learning (e.g., seminars) have been included as coaches' sources aiming to acquire new information and develop the knowledge and skills associated with quality coaching (3, 4). Apart from the key competencies of coaches (e.g., planning, organizing, and delivering training programs, working on sport-specific skills, and providing the correct verbal instructions), more recent approaches to the coach development process place emphasis on creating learning pathways considering a psycho-social approach, entailing intrapersonal (e.g., self-awareness, and emotional regulation), and interpersonal knowledge (e.g., effectively communicate with parents, athletes, and stakeholders) (5, 6). Within the coaching environment, these domains can be shaped and developed through reflection, self-evaluation (7), and personal experiences (often characterized as episodic experiences) (8), as well as mentoring (9), and interaction with other coaches and peers (10).

To empower and encourage their athletes’ personal and performance excellence, coaches are suggested to follow an athlete-centered approach (11). According to a recent study, professional coaches from different countries seem to acknowledge the usefulness of this model of coaching in producing more skilled athletes (12). Such an approach is also associated with providing a positive and secure training environment in which life skills can be developed (13), taking into consideration the considerable influence of coaches on their athletes. Under certain conditions (14), life skills, such as time management, and nonverbal communication, can be successfully transferred and applicated in different contexts beyond sports (e.g., family, work) (15). In parallel, and consistent with the holistic development of athletes through sports participation characterizing the athlete-centered approach (16), minimizing the potential negative consequences of early sports specialization has gained increased attention in the past two decades (17). In this direction, a number of models for athletes' long-term development (e.g., the Long-Term Athletic Development) (18), have been established and implemented to support sports performance through a healthier future career pathway.

Coach learning and development has been a topic of great interest, analyzing different learning situations and skills with the ultimate goal to support coaches' efficiency during daily practice (19), while the related research has focused on a variety of individual and team sports (20). In some of the initial studies, interpersonal knowledge, in terms of examining the influence of other elite coaches and mentors, was included as the primary resource for learning the skill of coaching (21, 22). In a more recent approach, Sherwin et al. (23) analyzed the learning resources of team sports coaches in Ireland. In this case, self-directed learning (e.g., reflective practice, previous experience, and interaction with peers), was reported as the most important factor facilitating the learning process, while in parallel, the importance of formal coaching was minimized. These findings are supported by the study of McIlroy (24) in a sample of over 3,700 coaches in the United Kingdom who reported that “talking to other coaches” was the most influential source of learning. In the case of individual sports, coaching creates different demands, compared to team sports, for example, cooperation with teammates during competitive situations (25). In the study of Irwin et al. (26), 16 elite-level men's artistic gymnastic coaches were interviewed, highlighting the role of individuality during the learning process as well as the importance of experiential learning and reflective practice during coaching development.

Swimming is predominantly an individual sport, where success at the highest level requires large amounts of physical and mental effort (27), in conjunction with specific anthropometrical, biomechanical, and technical requirements (28). In addition, swim coaches have to deal with four specific strokes, across multiple events (of varying distances). Considering that competitive swimming is an area of great research interest, it is not surprising that a series of studies have been conducted to analyze the perceptions of competitive swim coaches, mainly from training and physiological perspectives, such as resistance training practices (29), competition preparation (30), warm-up protocols (31), and recovery strategies (32), all of which may serve as guidelines for daily practice. Particular attention has also been given to the analysis of swimming performance (33), emphasizing the important role of technical ability during water movement aiming to improve the overall swimming efficiency. On the contrary, swim coaches' beliefs related to psycho-social issues have been underrepresented in the existing literature. Among these related studies, Callary et al. (34) analyzed the responses of a group of Masters swim coaches, demonstrating six main learning sources, including formal education, informal education, and coaching experiences. In addition, Junggren et al. (35) examined coaching practices and philosophy specifically related to the Danish high-performance swimming environment, while Brackley et al. (36) reported specific perspectives of skill acquisition for freestyle swimming in a sample of 20 elite Australian coaches. Consequently, looking more closely at the existing literature, the need to further investigate psycho-social issues and training practices together can provide a greater understanding of the swimming coaching process.

Toward a more holistic approach to sports coaches' learning, professional development, and training practices, it is also important to consider specific characteristics namely coaching experience, educational level, and gender as these may influence coaching behavior and efficacy (37, 38). Indeed, a previous study has reported the perception of higher competency of more experienced coaches (when compared to their novice counterparts) (20), while others highlighted the impact of educational qualifications on coaching practices (39), and emphasized the importance of gender on the coach-athlete relationship (40). Yet, it is only the study of Mesquita et al. (41), in a group of coaches of both individual and team sports, that analyzed sports coaches' perceptions of learning sources as related to the combination of their professional background and gender. Although the above-mentioned study has provided valuable insights for coaches in general, to date, this kind of information is lacking in swimming. Moreover, coaches' learning pathways have been transformed as social media integration has increasingly affected the coaching process (42). Extending the existing research by exploring coaching experience, educational level, and gender together, can bring a greater understanding of coaches' contemporary perceptions related to the overall training process, and serve as information to coach educational programs, with the final aim to potentially improve the effectiveness of coaching in swimming. Considering coach experience, educational level, and gender, this study aimed to explore the perception of swim coaches regarding their professional development and current training practices, with a particular emphasis on performance analysis.

## Materials and methods

### Participants

To estimate the sample size, an *α priori* power analysis was conducted using G*Power (v. 3.1.9.7) (43) to determine the minimum sample size required. Using Cohen's d (44) criteria, results indicated the required sample size to achieve 95% power for detecting a medium effect size of d = .30, at a significance criterion of *α* = .05, was 122 subjects. Finally, a total of 123 swim coaches (96 male/27 female) of competitive swimmers fully completed an online anonymous self-administered survey, in English only (https://survey.auth.gr/index.php/338556?newtest=Y&lang=en) (LimeSurvey Open- Source platform, GmbH, Hamburg, Germany). Initially, 180 responses were received from the 15th of January to the 24th of April 2022; however, 57 responders (32%) were excluded from the final analyses due to not completing >85% of the survey questions (45). The inclusion criteria defined responders to be currently active swim coaches and have established a career in coaching (i.e., working with national or elite-level swimmers). Four groups were considered according to the responders' coaching experience (in years), following the 10-year-rule for the attainment of expertise in sports coaching (46): (i) intermediate (up to 10 years) (ii) experienced (from 11 to 21 years), (iii) highly experienced (from 22 to 33 years) and very highly experienced (34 years or more). In addition, as the education/qualification level can influence coaching competencies (20), the responders were grouped as follows: (i) studied sports science at a third-level institution (University), (ii) with no studies related to sports science, and, (iii) with qualification through a national coaching certification programme or from a private organization.

### Survey design

In this descriptive study, the survey was developed according to the aim of the study, with no leading hypothesis, and was reviewed, towards improving clarity and usability, by three external swim coaches (not included in the sample) of swimmers competing at the Olympic Games, two of whom held Ph.D. degrees in sports science. Furthermore, a pilot study was conducted with two academic colleagues with relevant backgrounds in survey design, as well as two swimming team staff to evaluate the content validity of the survey (i.e., determine any possible respondent fatigue, improve technical terminology, and test for accuracy) for the targeted population (29). Finally, the survey was sent for a trial analysis. According to the feedback provided, two more questions were added, three questions were removed, and five questions were rephrased for better understanding. Therefore, the final survey contained 36 questions, all of them qualitative in nature, with four specific types (closed-ended using the Likert scale, yes/no, and multiple-choice questions), categorized into six sections as follows: (1) *Background Information*, (2) *Developing Swim Coaching Through Learning*, (3) *Self-evaluation*, (4) *Interpersonal-Intrapersonal Interactions*, (5) *Life skills*, (6) *Analyzing Swimming Performance*. The final section included an open-ended question asking the coaches to express themselves freely for additional comments or remarks. The introductory page of the survey included a weblink with a concise description, explanation, and all the necessary information, along with the General Data Protection Regulation statement (GDPR), and the informed consent with a yes/no style question.

### Procedures

The survey was distributed via email, personal contacts, social media, and the World Swim Coaches Association. The average time taken by coaches to complete the survey was approximately 15 min. Ethical approval was provided by the first author's institutional Ethics Review Board (30/8-3-2022) in advance of the survey distribution and conformed to the Declaration of Helsinki.

### Statistical analysis

The survey responses were processed anonymously and initially exported to Microsoft Excel for descriptive analysis. To validate the assumptions of normality, the Kolmogorov-Smirnov test was applied, indicating a parametric distribution of the data (*p* > 0.05). To ensure internal consistency Cronbach's alpha was assessed for each subsection, showing acceptable alpha values (ranging from 0.76 to 0.84). Pearson chi-square test (*χ*^2^) was used to examine the association between coaches’ perceptions and beliefs, and their experience, educational level, and gender. The frequency of answers was categorized as follows: all represented 100% of relevant participants; most indicated ≤75%, majority referred to 55 to 75%, approximately half denoted ∼50%, approximately a third indicated ±30%, and minority represented >30% (47). All Statistical analyses were performed using IBM SPSS (version 26; SPSS Inc, Chicago, IL). The alpha value was set at p* *≤ 0.05.

## Results

### Coaches' characteristics

The survey responders were spread across 41 countries, with the largest response coming from the United States (*n* = 52; 42.3%), followed by Australia (*n* = 10; 8.1%). In general, 24 responders originated from Europe, 65 from America, 19 from Asia, four from Africa, and 11 from Oceania (Australia and New Zealand). The distribution of responders by age group indicated that approximately a third belonged to the 55 + group (*n* = 45; 35.7%), followed by the 46–50 and 41–45 groups (*n* = 17; 13.5%, and *n* = 16; 12.7%, respectively). The average coaching experience was 25.1 ± 13.4 years, with the average number of athletes under their supervision at the highest level of 14.3 ± 19.5. Regarding the competition level of the athletes, 80.2% participated in or were preparing for National Championships, 22.2% for Continental Championships, and 27.0% for World Championships and/or Olympic Games trials.

### Developing swim coaching through learning

**Figure 1:**
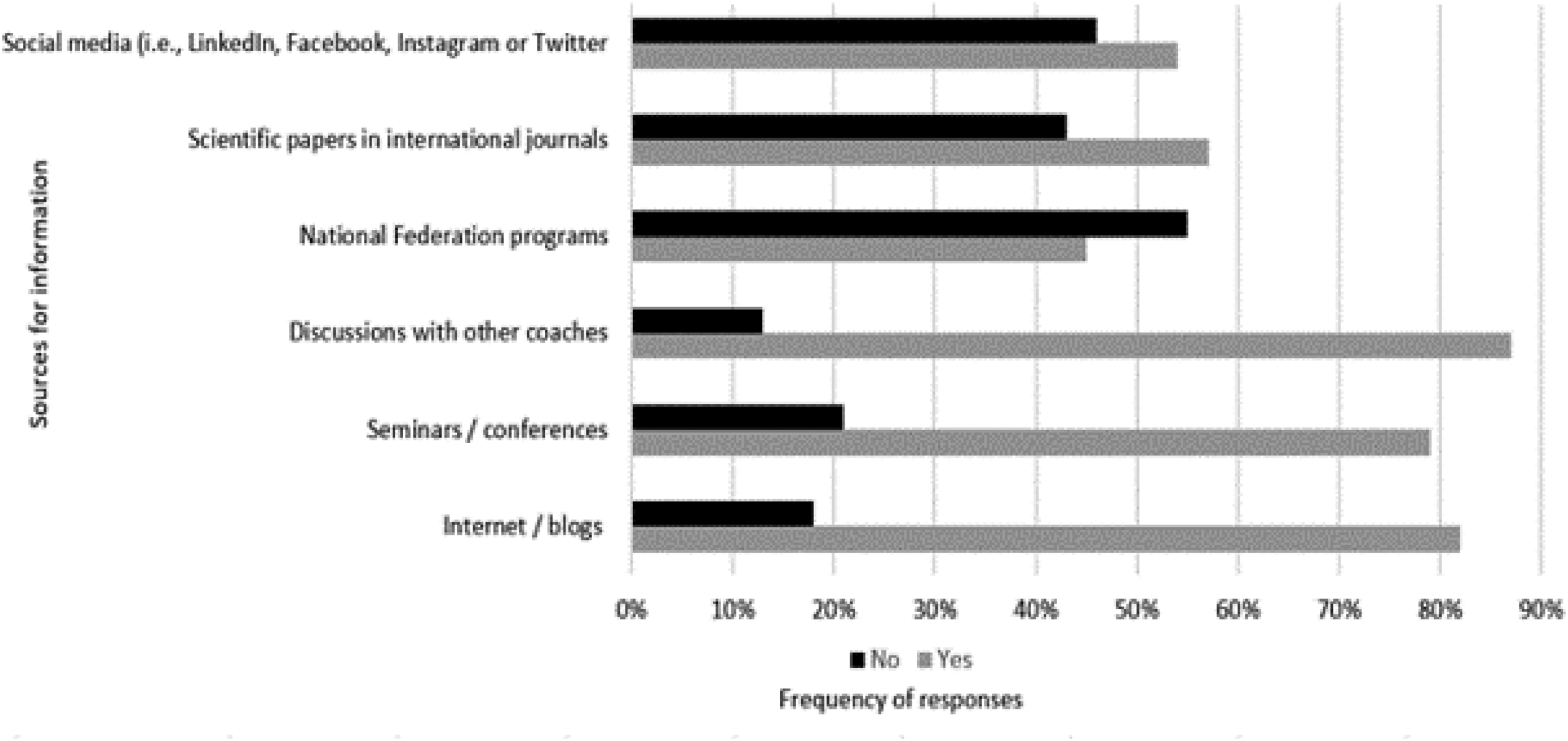
Sources of information for coaches regarding new developments and trends in swimming coaching.

Less than half of the responders studied sports science at a third-level institution (*n *= 52; 42.3%). Thirty-eight responders (30.9%) did not have studies related to sports science. The remaining responders attained their qualification through their respective national coaching certification programme, or from a private organization (*n* = 33; 26.8%). Most of the responders reported that they were regularly informed about new developments and trends in swimming coaching (*n* = 117; 95.1%), while the most common sources of information included discussions with other coaches (*n *= 104; 88.9%), followed by internet/blogs (*n *= 95; 81.2%), and seminars/conferences (*n *= 92; 78.6%) (Figure 1).

Coaches were also asked if they consider formal education as a prerequisite in competitive swimming. Sixty-five responders (52.8%) selected the predefined answer “education is a lifelong learning process and never stops”, 30 coaches (24.4%) answered “yes”, and 16 responders (13.0%) answered “no”. Of the remaining responders, 12 (9.8%) chose the answer “It depends on the coach”. Most of the responders (*n* = 99; 80.5%) reported implementing the principles of Long-Term Athletic Development [LTAD] in their annual training program. Of those responders implementing the LTAD model, 87 coaches (87.9%), reported that they consider this model useful and/or easy to implement.

### Self-evaluation

Most of the responders (*n* = 104; 85.2%) reported that they still have the same passion to improve their coaching skills as they had in the initial stages of their careers. Coaches were then asked if their coaching philosophy has changed over the years. The majority (*n* = 78; 63.4%) reported selecting the predefined answer “yes, it has been changed as a result of the knowledge and experiences I have acquired”. Twenty-one responders (17.1%) reported that “yes, it has been transformed through” “episodic experiences”. Of the remaining responders, the answer “yes definitely, it has been adapted to the demands and requirements of the job” was the choice of 20 responders (16.3%). Finally, four responders (3.3%) reported maintaining the same coaching philosophy. The majority of the responders (*n* = 78; 63.4%) reported reflecting on themselves, mainly after every competition (*n* = 79; 64.2%).

### Life skills

**Figure 2:**
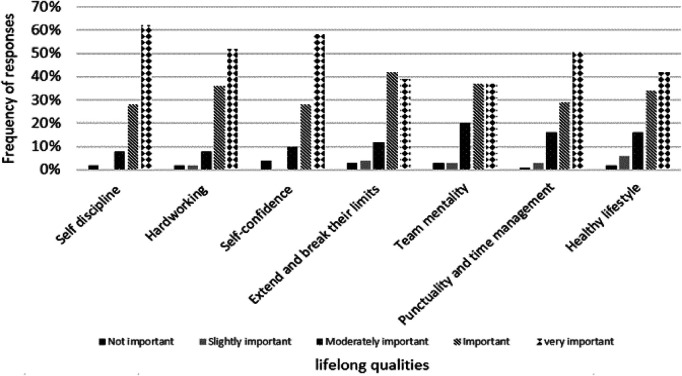
Ranking of the perceived most important standard skills for a “good” swimming coach.

Most of the responders (*n* = 89; 80.9%) reported that they had a mentor during their coaching career. Of those, 76.5% (*n* = 65) stated that the impact of this mentor was extremely influential on their development as swimming coaches, while the rest of the responders reported a moderate influence (*n* = 20, 23.5%). No responses were returned for either “slightly influential” or “not at all”. The last question in this section required the participants to rate what they consider the most important lifelong qualities their athletes have gained from their influence, and the sport itself. Self-discipline (*n* = 76; 62.3%), and self-confidence (*n* = 72; 58.4%) were the two most important life skills according to the responders (Figure 2).

### Interpersonal and Intrapersonal Interactions

**Figure 3:**
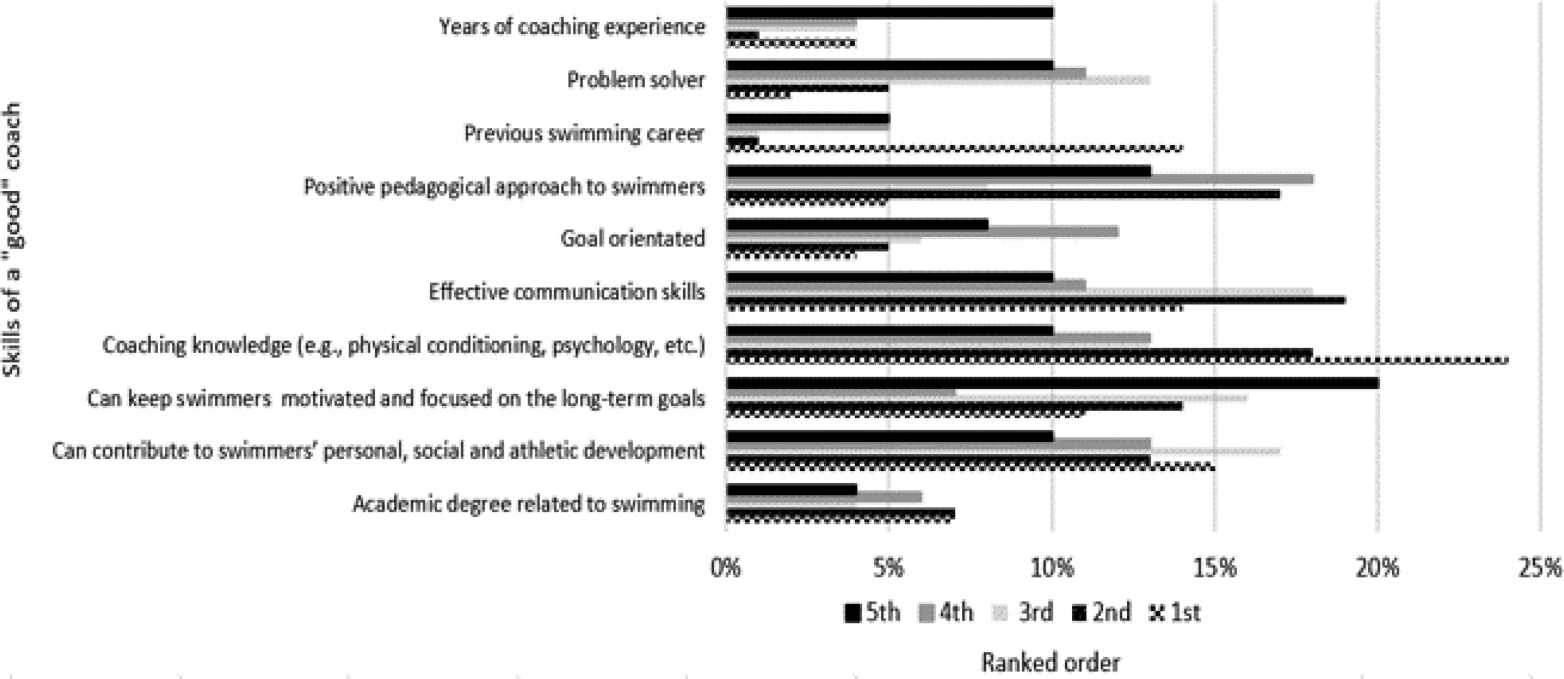
Ranking of the perceived most important lifelong qualities.

Most of the responders reported having an assistant coach (*n* = 86, 81.9%). In most cases (*n* = 68; 79.1%), the decision of who the assistant coach was, was made by the responder. The responders stated that they exchange knowledge and experiences with coaches from other clubs/programs (*n* = 95; 90.5%). Of these, approximately half (*n* = 50; 52.6%) answered that they engage in this knowledge/experience exchange “often”, while the remaining responders chose “sometimes” (*n* = 45; 47.4%). Most of the responders (*n* = 108; 92.3%) reported that they have support and understanding from their family and friends regarding their working hours during training, competitions, and training camps. Finally in this section, coaches were asked to rate, from most to less important, specific standard skills, that a “good” coach must have in his/her knowledge portfolio. Coaching knowledge, in terms of topics including physical conditioning and psychology, received overall the higher average ranking (24.5%) (Figure 3).

### Analyzing swimming performance

In the first question of this section, coaches were asked if they use any software, analytical methods, or models to try and predict the competitive performance of their swimmers. Approximately half of the responders (*n* = 55; 55.6%) reported using such software/systems, with responders appearing to apply these methods/models during training camps (*n* = 16; 16.2%), or under the guidance of their swimming federation/with the help of sport scientists (*n* = 12; 12.1%). Following this, coaches were asked about their familiarization with multiple analysis applications. Approximately a third of the responders (*n* = 32; 32.3%) answered “somewhat familiar”, while almost the same number of responders chose the option “familiar” (*n* = 30; 30.3%). Of the remaining responders, the answer “not familiar” was the choice of 24 responders (24.2%), and 13 responders (13.1%) answered “very familiar”.

**Figure 4:**
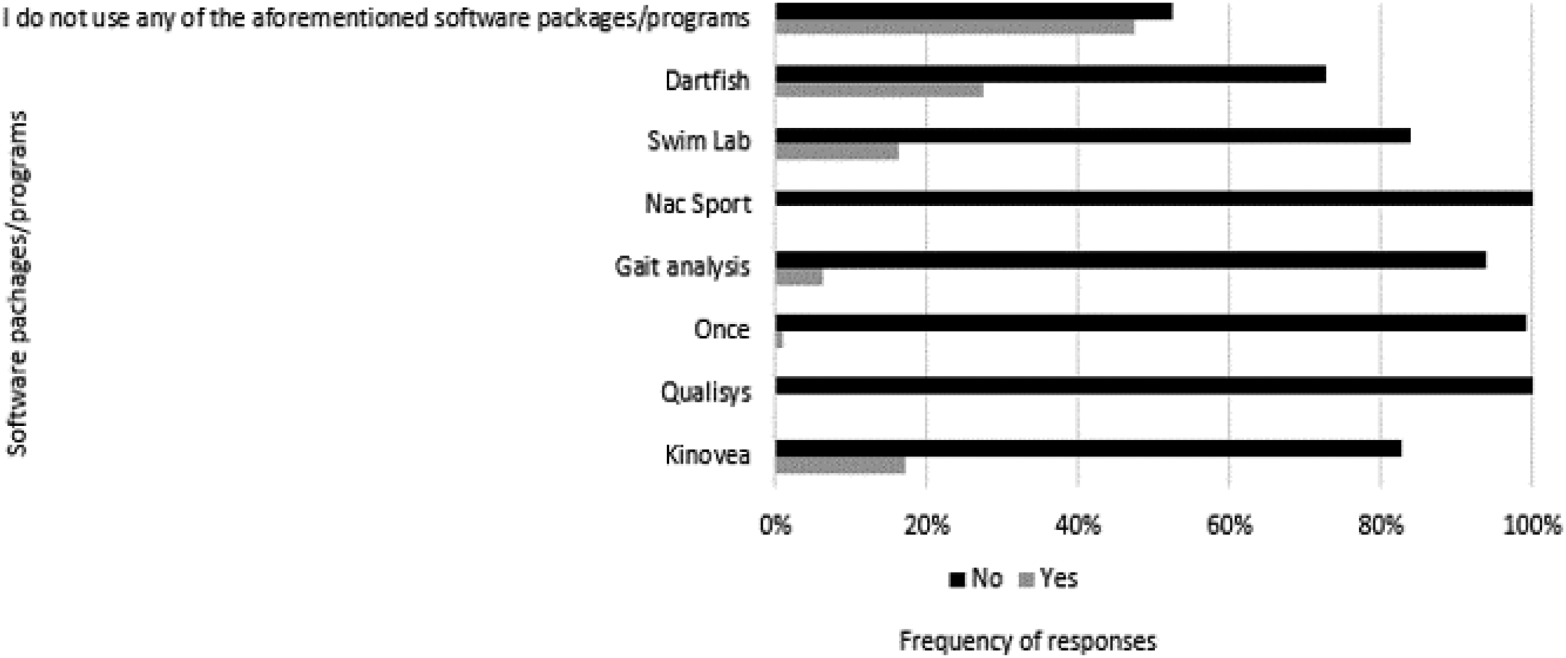
The use of specific software packages/programs for performance analysis.

Coaches were then asked to respond about the use of specific software packages/programs/wearables for performance analysis. The data presented in Figure 4 indicates that approximately half of the responders (*n* = 52; 52.5%) do not use such programs or devices, while for those that do use them, Dartfish was the most commonly used (*n* = 27; 27.3%). This question included the option “other” with participants mentioning additional devices and software such as “Commit Swimming” (*n* = 3) and “TritonWear” (*n* = 5).

**Figure 5:**
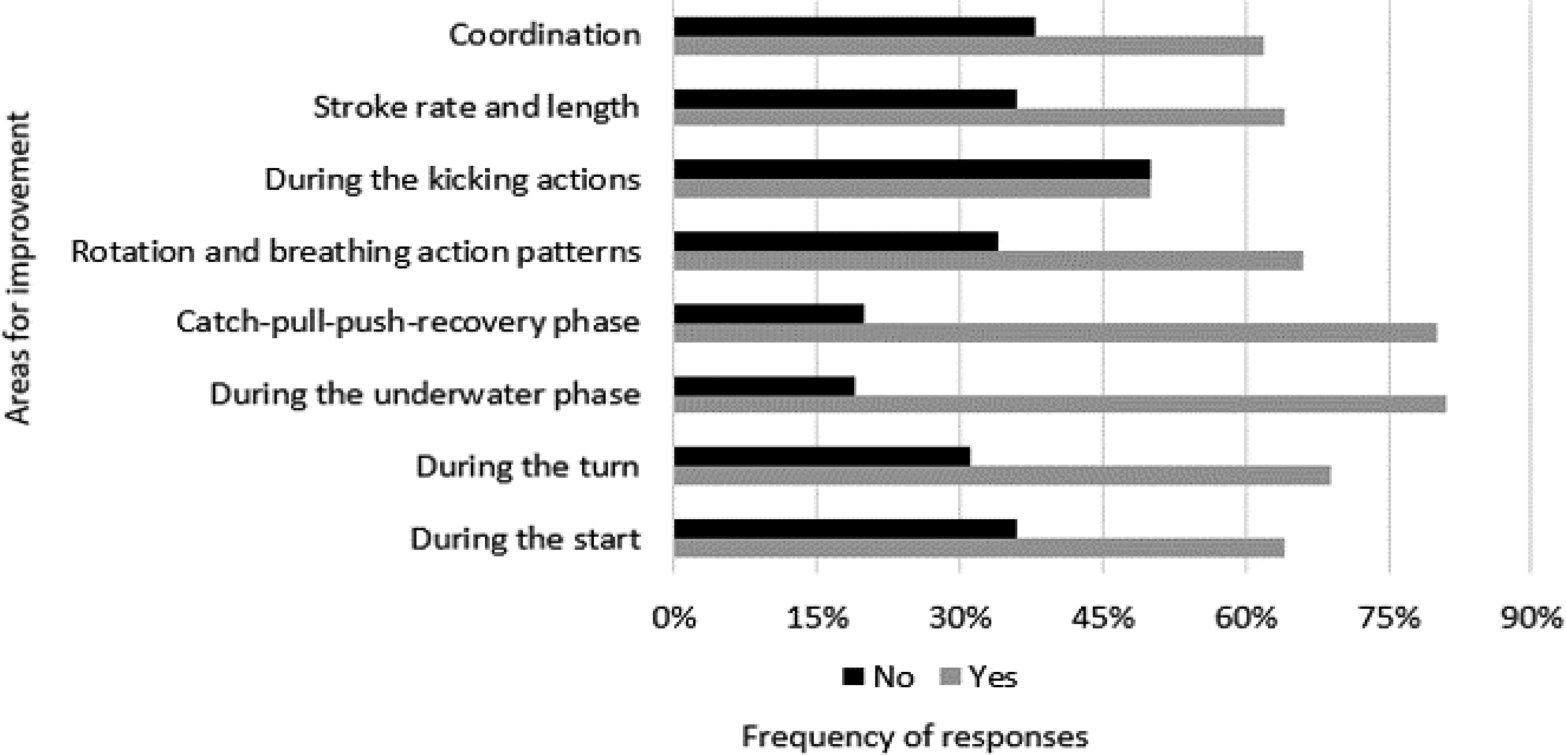
Areas of technical and tactical improvement obtained during video analysis.

Approximately half of the responders (*n* = 46; 46.5%) seem to focus more on performance indicators (i.e., starts, turns, and splits) when analyzing the data obtained during video analysis, while the remaining responders give more attention to kinematic parameters (e.g., stroke length) (*n* = 30; 30.3%), and kinetic parameters (e.g., force) (*n* = 12; 12.1%). Eleven responders (11.1%) answered that they do not use video analysis. Those who reported using of video analysis of their athletes, were also asked to report the most frequent area for technical and tactical improvement. Swimmers' underwater phase and swimming stroke kinematics (catch-pull-push) were the two most common answers (*n* = 81 and 79; 81.8% and 79.8%, respectively), with other areas also being highly reported (Figure 5).

**Figure 6:**
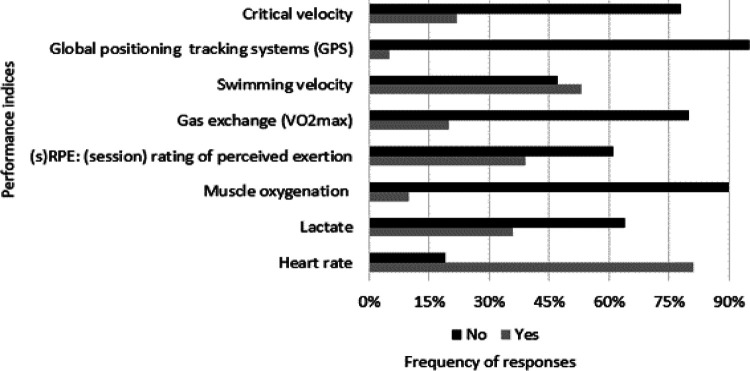
Performance indices used by coaches to track the performancer/progress of their athletes, excluding race performance (i.e., final time or position).

Heart rate was the performance index mostly used by the responders (*n* = 82; 82.8%) when race performance was excluded. Other popular indices included swimming velocity (*n* = 53; 53.5%), rate of perceived exertion (RPE) of each training session (*n* = 36; 36.4%), and lactate (*n* = 31; 31.3%) (Figure 6).

When asked to report the training intensity distribution or the periodization model coaches use to prescribe training intensity, the responders mostly declared that they combine different periodization models, depending on the phase of the competitive season (*n* = 60; 60.6%) as well as according to the primary event of their athletes (i.e., sprint, middle-distance, or long-distance) (*n* = 53; 53.5%). Further, the minority of the responders reported the use of the “threshold” (*n *= 24; 24.2%), “polarized” (*n *= 20; 20.2%), and “pyramidal” (*n *= 13; 13.1%) intensity distribution or periodization models to prescribe training intensity.

Finally, coaches were given the opportunity, in their own words, to report any kind of boundaries or restrictions that they met during their coaching career. Valuable insights were gained on this topic, including gender inequality in coaching, a lack of pool space, time limitations, administration issues, and financial issues. The impact of the responders' coaching experience, educational level, and gender on their perceptions and coaching practices are presented in Tables 1–3, respectively. Due to the large number of questions in the survey, only those that revealed statistical significance are shown.

**Table 1:** Percentage and statistical analysis according to the responder's coaching experience.

Responder's experience level				*χ*^2^ (df)	*p*
Questions					
In which way are you regularly informed about new developments and trends in swimming coaching? (Seminars)					
Experience level (years)	Yes	No	N/A	13.276 (6)	0.039
0–10	55.0%	45.0%	0.0%		
11–21	68.6%	22.9%	8.6%		
22–32	88.2%	8.8%	2.9%		
33+	79.4%	14.7%	5.9%		
Was your assistant coach your choice?					
	Yes	No	N/A	14.921 (6)	0.021
0–10	45.0%	15.0%	40.0%		
11–21	37.1%	25.7%	37.1%		
22–32	58.8%	11.8%	29.4%		
33+	79.4%	5.9%	14.7%		
Do you exchange knowledge and experiences with coaches from other clubs-programs?					
	Yes	No	N/A	13.571 (6)	0.035
0–10	55.0%	25.0%	20.0%		
11–21	71.4%	8.6%	20.0%		
22–32	85.3%	2.9%	11.8%		
33+	88.2%	2.9%	8.8%		

N/A, No answer; χ^2^, Pearson chi-square test; (df), degrees of freedom; *p*, the alpha value was set at *p* ≤ 0.05.

**Table 2:** Percentage and statistical analysis according to the responder's educational level.

Responder's educational level				*χ*^2^ (df)	*p*
Questions					
In which way are you regularly informed about new developments and trends in swimming coaching? (Seminars)					
Educational level	Yes	No	N/A	12,869 (4)	0.012
No studies	57.9%	34.2%	7.9%		
At a 3rd level institution (University - College)	88.5%	11.5%	0.0%		
At a coaching/GNC programme	72.7%	18.2%	9.1%		
In which way are you regularly informed about new developments and trends in swimming coaching? (Federation programmes)					
	Yes	No	N/A	11.094 (4)	0.026
No studies	28.9%	63.2%	7.9%		
At a 3rd level institution (University - College)	44.2%	55.8%	0.0%		
At a coaching/GNC programme	57.6%	33.3%	9.1%		
In which way are you regularly informed about new developments and trends in swimming coaching? (Scientific papers in International journals)					
	Yes	No	N/A	16.047 (4)	0.003
No studies	34.2%	57.9%	7.9%		
At a 3rd level institution (University - College)	73.1%	26.9%	0.0%		
At a coaching/GNC programme	48.5%	42.4%	9.1%		
Was your assistant coach your choice?					
	Yes	No	N/A	13.164 (4)	0.011
No studies	52.6%	15.8%	31.6%		
At a 3rd level institution (University - College)	63.5%	21.2%	15.4%		
At a coaching/GNC programme	48.5%	3.0%	48.5%		
Do you exchange knowledge and experiences with coaches from other clubs-programs?					
	Yes	No	N/A	13.532 (4)	0.009
No studies	76.3%	7.9%	15.8%		
At a 3rd level institution (University - College)	90.4%	5.8%	3.8%		
At a coaching/GNC programme	57.6%	12.1%	30.3%		
Do you implement the principles of the “Long Term Athletic Development (LTAD) when you plan the annual training program?					
	Yes	No		7.587 (2)	0.023
No studies	65.8%	34.2%			
At a 3rd level institution (University - College)	86.5%	13.5%			
At a coaching/GNC programme	72.1%	27.9%			

N/A, No answer; GNC programme, Government's national coaching certification programme; χ^2^, pearson chi-square test; (df), degrees of freedom; *p*: the alpha value was set at *p* ≤ 0.05.

**Table 3:** Percentage and statistical analysis according to the responder's gender.

Responder's gender					*χ*^2^ (df)	*p*
Questions						
Do you consider formal education as a prerequisite for coaches in high-level competitive swimming?						
Gender	Yes	No	It depends on the coach	Education is a lifelong learning process and never stops		
Male	26.0%	13.5%	6.3%	54.2%	13.379 (6)	0.037
Female	19.2%	11.5%	19.2%	50.0%		
How familiar are you with multiple analysis applications?						
	Very familiar	Familiar	Somewhat familiar	Not familiar		
Male	12.8.%	34.6%	35.9%	16.7%	13.744 (6)	0.033
Female	15.0%	10.0%	25.0%	50.0%		

χ^2^, pearson chi-square test; (df), degrees of freedom; *p*, the alpha value was set at *p* ≤ 0.05.

The education level of the responders significantly influenced their familiarity with software, analytical methods, or models to predict the competitive performance of their swimmers (*χ^2 ^= *20.822; *p = *0.02). Moreover, all answers related to the indices used to track the performances/progress of swimmers were affected by the educational level (*χ^2 ^= *11.535 to 16.664; *p = *0.021 to 0.002). Similarly, this variable impacted the main training intensity distribution/periodization model applied to prescribe training intensity (*χ*^2^ = 9.061 to 13.770; *p* = 0.045 to 0.008), except for the answer “combination of models”.

## Discussion

This study aimed to explore swim coaches' perceptions regarding their professional development and current training practices, with a focus on performance analysis, by considering their coaching experience, educational level, and gender. Through an online survey, swimming coaches from different countries were able to share their views, allowing for valuable insights to be garnered. Notably, the results showed that the responders' educational level is likely to have a larger impact on the parameters affecting the training practices analyzed. The high quality of participants surveyed in this study is demonstrated by the fact that 27% of responders were working with swimmers preparing for the upcoming World Championships and/or Olympic Games trials.

The importance of pursuing professional knowledge and staying up to date was emphasized, as almost all responders (95.1%) reported that they are regularly informed regarding new developments and trends in swimming coaching. The learning sources mainly selected by the responders (i.e., internet searches and other coaches) are in agreement with those reported in previous studies on coach learning related to swimming (34), and a variety of individual and team sports (4). Interestingly, half of the responders considered formal education a requirement for coaching in competitive swimming. The majority of the responders (87.9%) in this study seem to have similar viewpoints regarding the usefulness of the LTAD model during daily practice, even though this model is probably not a part of all national certification programs. This finding is consistent with that of Costa et al. (48), who reported similar acceptance during daily practice (∼83%) in a group of 87 swim coaches, independent of their experience level. In any case, the usefulness of the LTAD model, adjusted to daily swim practice, certainly has its strengths and weaknesses, for example, the excessive emphasis on training volume instead of technique development. In parallel, the need for more longitudinal data analysis related to the implementation of long-term development models in swimming has previously been highlighted (49).

### Professional development

According to almost all of the responders (96.7%), their coaching philosophy has changed over time. This procedure seems to be normal, as it is related to reflection, coaching, and life experiences (50). In a recent study of a sample of swim coaches, participants reported to relying largely on their coaching philosophy during decisions making for issues related to the technical analysis of their athletes (33). Meanwhile, although the existing body of literature has predominantly focused on the importance of formatting coaching philosophy, its origins, and operation (51), bridging the gap between coaching philosophy in theory and in practice remains critical for expertise in sports coaching (52).

According to the results of the current study, it is apparent that coaching knowledge (in terms of physical conditioning, and sports psychology) was ranked as the primary characteristic to become a “good’ swimming coach” (22.2%). This finding is surprising given that communication skills were not among the top-rated standard skills (10.3%), as the existing body of literature related to expertise in coaching gives a lot of attention to communication as a “fuel” to support interpersonal skills, resulting in positive performance-related outcomes (53), as well as high levels of satisfaction within sports (54). While it is important to define the key characteristics of successful coaching to ensure competitive athletes' success, the wide range of areas involved in this procedure (e.g., coaching skills, coaching knowledge, and psychological factors) makes it even more challenging to be explored (55).

Although not clearly defined, mentoring is described as a highly effective way to learn how to coach (56), while it is probably experienced as a positive and useful process by those involved, especially during their first years of coaching (57). Most of the responders surveyed here (80.9%) reported the existence of a mentor during their coaching career, supporting the findings of Lemyre et al. (58), with a clear impact on their coaching development as swimming coaches (76.5%). This facilitative relationship between a coach and their mentor has primarily been viewed as a practical learning experience, emphasizing implementing effective mentoring to support coaches' development (59). In the same context in which social interaction is involved, coaches' learning through collaboration and discussion with peers has been suggested as a critical approach to facilitate professional development (6). The majority of responders surveyed here (90.5%) reported exchanging knowledge and experiences with coaches from other clubs/programs, although this procedure seems to not be frequently applied. For instance, this result is in contrast to the findings of Lemyre et al. (58) in a group of youth sports coaches from different sports, implying an “unwritten rule” describing the unwillingness to share nothing more than the usual information related to daily practice. However, in a more recent study on competitive swimming coaches (33) “discussion with other coaches” was presented as a highly ranked source of information affecting the implementation of performance analysis methods.

The list of lifelong qualities (life skills), that under the right conditions can be developed through sports (60), is large and it has previously been divided into distinct categories, namely commitment, positive values and identity, and social competencies (61). In the current study, among seven predefined answers, the responders selected self-discipline and self-confidence as the two most important lifelong qualities that athletes have gained from their influence, and the sport itself. On the contrary, team mentality was the answer mostly viewed as not important, probably due to the nature of swimming as an individual sport. Certainly, the process of life skills learning and transfer outside the sports context requires the application of deliberate strategies by sports coaches, complementarily to creating opportunities for athletes to do so (14).

### Training practices

The use of software, analytical methods, or models for performance prediction was apparent to over half of the responders (55.6%), with the familiarization rate related to multiple analysis applications being less than that (43.4%). This lack of familiarization with technology was also reported in a previous study by Mooney et al. (33) in a large group of swim coaches. Therefore, it seems that the responders surveyed in the current study may rely more on “naked-eye” observations and not on information from quantitative analysis, indicating that implementing technology into daily practice still remains a challenge. Subsequently, although the need to support collecting performance analysis information is increasing, swim coaches continue to rely on their instinct and experience to evaluate and understand competitive performance. To resolve this issue, collaborative relationships between coaches and sports scientists, or accessing more sophisticated tools through nationally coordinated programmes/swimming federations are recommended (33, 62). For those that reported the use of performance analysis programs, Dartfish and Kinovea were the two most commonly used, and, in those cases, performance indicators (i.e., starts, turns, and splits), and kinematic parameters were the focus points. This finding reflects previous research that proposed the importance of “temporal parameters” (e.g., stroke rate and splits) for swim coaches during swimming analysis (33). In addition, the underwater phase, and swimming stroke kinematics were the most chosen areas for technical improvement.

The high preference obtained among the responders (82.8%) regarding the use of heart rate as a performance index can be explained by its practical value as a simple, and cost-effective tool with an increasing number of available software for data analysis (63), even though only a limited number of performance-related information can be derived from it (64). In addition, the rest of the performance indices mostly preferred by the responders (i.e., RPE and lactate) were in agreement with the results of similar research related to monitoring practices in swim coaches (65, 66). Nevertheless, such indices may have a lesser value when considered in isolation, as the combination of different testing procedures may offer a more comprehensive evaluation of swimmers' training status (67).

Finally, the responders' preference regarding the periodization model applied based on the primary event of their swimmers (53.5%) was also highlighted in a recent systematic review of elite swimmers (68). However, the most common approach reported (60.6%) was the combination of different periodization models listed in this survey, depending on the phase of the competitive season (i.e., “threshold”, “pyramidal”, and “polarized”). In any case, these two answers could be considered interchangeably as swim coaches' seasonal training practices are highly dependent on athletes' individual needs and characteristics.

### Effect of coaching experience, educational level, and gender

The results concerning the impact of experience level suggested that the 22 to 33 years category (years of coaching experience) preferred seminars as a source of information about new developments and trends in swimming. In addition, it becomes clear that the responders with the highest experience level (33 + years) were more open to sharing knowledge with coaches from other clubs. Indeed, according to the review study of Walker et al. (69), the existing literature on learning sources of sports coaches, emphasizing the more experienced ones, revealed that independent learning and interaction with other coaches appear to be the most common types of learning. Moreover, empirical evidence suggests that more experienced coaches have developed self-confidence during their professional growth, including a willingness to share their knowledge and expertise with other coaches. As their approach to how they coach (e.g., training philosophy) has been developed and refined over a number of years, potentially in response to success, setbacks, etc., they have ended with an approach that works for them and are happy to share and articulate that to their peers.

More insights into coaching development can be derived through the results regarding the educational level of the responders. For instance, it was found that those who hold a third-level degree are more likely to implement the principles of the LTAD model during their annual training plan. However, it is possible that the LTAD framework may not be introduced into the education programs of many NGB qualifications globally. Moreover, these responders appear to rely more on scientific papers to be informed about new developments and trends in swimming. This finding was somewhat expected since the “academic language” is likely poorly understood by coaches with no academic education (20, 70), in addition to the limited access to scientific journals and the difficulty to disseminate research findings (71). In fact, the necessity to translate and implement scientific knowledge into daily sports practice has been a topic of interest for a number of decades (72). In this sense, publishing research directly linked to specific sports (70), and challenging more applied research questions may facilitate the knowledge transfer of sports science to the coaching population (73).

The responders' gender did not seem to play an important role regarding the psycho-social issues and training practices analyzed in this study. Nevertheless, an interesting result was that male coaches tended to have greater familiarity with multiple analysis applications used for performance analysis in swimming. Previous evidence suggests that female coaches are underrepresented in high-level coaching, while often experiencing sexism and discrimination within the coaching environment (74, 75). To the authors' knowledge, this is the first study that has revealed a gender disparity in applying technology to swimming performance analysis.

The primary strength of the current study is that it provides an in-depth description and an up-to-date landscape of psycho-social perceptions related to professional development, as well as daily training practices, with a consideration of the professional background and gender of swim coaches of competitive athletes, from a number of different countries. However, certain limitations need to be acknowledged. Firstly, we recognize a possible response bias, since the largest proportion of the responders were from the United States, possible stereotypes, and cultural differences among the responders may have an impact on the results (76). For instance, the opportunities to attend seminars or to enhance knowledge of recent technology applied to swim practice may be limited to certain countries. However, as the majority of the responders were from Western countries, differences may be negligible. In any case, in this study, an effort was made to use precise and simple language and keep questions short, with a variety of options. In addition, it needs to be acknowledged the varying degree of emphasis that each country places on the sport. Secondly, as a large number of closed-ended questions were included, the authors cannot be sure that the responses given fully represent the current perception of those included. Thus, future research should consist of mixed methods, combing surveys, observational research methods, and interviews/focus groups to elicit further insights. Thirdly, despite the fact that the sample size was larger, when compared to previous research with an international approach in a single sport (30, 45, 65, 77), it can be considered relatively small compared to the range of the swim coaching population. As such, generalizing these findings may not be advisable. Finally, although the survey was piloted by a subset of coaches, not involved in the study, the test-retest reliability was not calculated. Despite these limitations, the results presented in the current study may be used for designing future coach education and development programmes, as they contribute to a deeper understanding of contemporary psycho-social and training perceptions of swim coaches, highlighting key issues related to their coaching experience, education level, and gender. With regards to how the findings from the current study could be used to inform the development of coach education programmes, it may be that such programmes should be offered in a tiered approach, dependent on the education level of the coach.

In conclusion, the current study provided an international perspective on swim coaches' professional development and a description of current training practices applied. In addition, useful insights regarding the effect of coaching experience, educational level, and gender on coaches' perceptions and preferences were provided, suggesting that educational level is one of the most influential factors when it comes to the training practices analyzed in this study. These findings clearly add a contribution to the existing literature on psycho-social issues and training practices involved in the competitive swimming environment.

## Data Availability

The original contributions presented in the study are included in the article/Supplementary Material, further inquiries can be directed to the corresponding author.
