# Supplementary material for: Swimming coaches’ professional development and training practices: an international survey
Source: Front Sports Act Living. 2023 Jul 18;5:1229066. doi: 10.3389/fspor.2023.1229066 (PMC10390787; doi:10.3389/fspor.2023.1229066)
Supplement: Supplementary file 1 [file Datasheet1.pdf]

Supplementary Material (ID: 1229066)

Statistical analysis according to the responder's *coaching experience*.

Are you regularly informed about new developments and trends in swimming coaching? (yes)

χ^2 = 2.40 (3); p = .493

If yes, then in which way? (internet)

χ^2 = 3.67 (6); p = .721

If yes, then in which way? (internet)

χ^2 = 6.18 (6); p = .403

If yes, then in which way? (federation)

χ^2 = 6.73 (6); p = .346

If yes, then in which way? (Scientific papers)

χ^2 = 4.76 (6); p = .575

If yes, then in which way? (Social media)

χ^2 = 10.68 (6); p = .099

Do you consider formal education as a prerequisite for coaches in high-level competitive swimming? **a**) yes t **b**) no **c)** it depends on the coach **d)** education is a lifelong learning process and never stops

χ^2 = 14.34 (9); p = .111

Do you implement the principles of the "Long Term Athletic Development" (LTAD) when you plan the annual training program? (yes / no)

χ^2 = 4.44 (3); p = .218

If yes, do you consider this model useful and / or easily implemented? (yes / no)

χ^2 = 9.30 (6); p = .157

Do you still have the same passion to improve your coaching skills as you had in the initial stages of your career? **a**) Yes a lot **b**) not that much **c**) No, I`m stuck & I need inspiration

χ^2 = 6.85 (6); p = .335

Has your coaching philosophy changed during the years? **a**) Yes definitely, it has been adapted to the demands and requirements of the job **b**) No, I maintain the same philosophy and destination in coaching **c)** Yes, it has been changed as result of the knowledge and experiences I have acquired **d)** Yes, it has been transformed through "episodic experiences" (e.g., interactions with a certain type of swimmer, a serious injury, family and/or personal commitment, success or failure, etc.).

χ^2 = 14.28 (9); p = .113

Do you have support and understanding from your family and friends regarding working hours and days, competitions, and training camps? (yes/no)

χ^2 = 3.30 (6); p = .770

Have you ever had a mentor during your coaching career? (yes/no)

χ^2 = 2.15 (6); p = .905

Do you have help from an assistant coach? (yes/no/sometimes)

χ^2 = 4.94 (3); p = .176

How frequently do you engage in this knowledge - experience exchange? (often/sometimes)

χ^2 = 4.37 (3); p = .933

Do you use any software, analytical methods or models to try and predict competitive performance of your swimmers? (a) Yes b) no c) sometimes under the guidance of your national swimming federation, and / or sport scientists d) during training camps where appropriate equipment is available

χ^2 = 7.51 (6); p = .276

Multiple analysis applications are available to help coaches with performance analysis specialized for swimming. How familiar are you with these methods? a) Very familiar b) Familiar c) Somewhat familiar d) Not familiar

χ^2 = 11.978 (9); p = .215

Statistical analysis according to the responder's *educational level*.

Are you regularly informed about new developments and trends in swimming coaching? (yes)

χ^2 = 4.67 (2); p = .097

If yes, then in which way? (internet)

χ^2 = 4.78 (4); p = .311

If yes, then in which way? (discussions)

χ^2 = 9.51 (4); p = .50

If yes, then in which way? (Social media)

χ^2 = 6.46 (6); p = .167

Do you consider formal education as a prerequisite for coaches in high-level competitive swimming? **a**) yes t **b**) no **c)** it depends on the coach **d)** education is a lifelong learning process and never stops

χ^2 = 10.29 (6); p = .113

If yes, do you consider this model useful and / or easily implemented? (yes / no)

χ^2 = 7.18 (4); p = .103

Do you still have the same passion to improve your coaching skills as you had in the initial stages of your career? **a**) Yes a lot **b**) not that much **c**) No, I`m stuck & I need inspiration

χ^2 = 3.19 (4); p = .526

Has your coaching philosophy changed during the years? **a**) Yes definitely, it has been adapted to the demands and requirements of the job **b**) No, I maintain the same philosophy and destination in coaching **c)** Yes, it has been changed as result of the knowledge and experiences I have acquired **d)** Yes, it has been transformed through "episodic experiences" (e.g., interactions with a certain type of swimmer, a serious injury, family and/or personal commitment, success or failure, etc.).

χ^2 = 9.41 (6); p = .152

Do you have support and understanding from your family and friends regarding working hours and days, competitions, and training camps? (yes/no)

χ^2 = 6.14 (4); p = .189

Have you ever had a mentor during your coaching career? (yes/no)

χ^2 = 8.56 (4); p = .073

Do you have help from an assistant coach? (yes/no/sometimes)

χ^2 = 2.35 (2); p = .309

How frequently do you engage in this knowledge - experience exchange? (often/sometimes)

χ^2 = 0.36 (2); p = .837

Statistical analysis according to the responder's *gender*.

Are you regularly informed about new developments and trends in swimming coaching? (yes)

χ^2 = 0.60 (2); p = .074

If yes, then in which way? (internet)

χ^2 = 4.78 (4); p = .310

If yes, then in which way? (seminars)

χ^2 = 3.33 (4); p = .504

If yes, then in which way? (discussions)

χ^2 = 5.44 (4); p = .245

If yes, then in which way? (federation)

χ^2 = 4.80 (4); p = .308

If yes, then in which way? (Scientific papers)

χ^2 = 2.17 (4); p = .703

If yes, then in which way? (Social media)

χ^2 = 3.65 (4); p = .455

Do you implement the principles of the "Long Term Athletic Development" (LTAD) when you plan the annual training program? (yes/no)

χ^2 = 4.94 (2); p = .084

If yes, do you consider this model useful and / or easily implemented? (yes / no)

χ^2 = 5.12 (4); p = .275

Do you still have the same passion to improve your coaching skills as you had in the initial stages of your career? **a**) Yes a lot **b**) not that much **c**) No, I`m stuck & I need inspiration

χ^2 = 0.75 (4); p = .944

Has your coaching philosophy changed during the years? **a**) Yes definitely, it has been adapted to the demands and requirements of the job **b**) No, I maintain the same philosophy and destination in coaching **c)** Yes, it has been changed as result of the knowledge and experiences I have acquired **d)** Yes, it has been transformed through “episodic experiences” (e.g., interactions with a certain type of swimmer, a serious injury, family and/or personal commitment, success or failure, etc.).

$\chi^2 = 2.42$ (6); $p = .877$

Do you have support and understanding from your family and friends regarding working hours and days, competitions, and training camps? (yes/no)

$\chi^2 = 3.25$ (4); $p = .510$

Have you ever had a mentor during your coaching career? (yes/no)

$\chi^2 = 2.36$ (4); $p = .670$

Do you have help from an assistant coach? (yes/no/sometimes)

$\chi^2 = 5.80$ (2); $p = .055$

If yes, was this coach your choice? (yes/no)

$\chi^2 = 7.00$ (4); $p = .136$

How frequently do you engage in this knowledge - experience exchange? (often/sometimes)

$\chi^2 = 1.16$ (1); $p = .203$

Do you use any software, analytical methods or models to try and predict competitive performance of your swimmers? (a) Yes b) no c) sometimes under the guidance of your national swimming federation, and / or sport scientists d) during training camps where appropriate equipment is available

$\chi^2 = 5.61$ (4); $p = .230$
